# Pyroptosis and Its Role in SARS-CoV-2 Infection

**DOI:** 10.3390/cells11101717

**Published:** 2022-05-23

**Authors:** Zsofia Agnes Bittner, Markus Schrader, Shilpa Elizabeth George, Ralf Amann

**Affiliations:** 1 Department of Immunology, Interfaculty Institute for Cell Biology, University of Tübingen, 72076 Tübingen, Germany; zsofibittner@gmail.com (Z.A.B.); shilpaelise@gmail.com (S.E.G.); 2 Department of Radiooncology, Marienhospital Stuttgart, 70199 Stuttgart, Germany; markusschrader001@gmail.com

**Keywords:** pyroptosis, gasdermins, NLRP3, SARS-CoV-2, interleukin-1β

## Abstract

The pore-forming inflammatory cell death pathway, pyroptosis, was first described in the early 1990s and its role in health and disease has been intensively studied since. The effector molecule GSDMD is cleaved by activated caspases, mainly Caspase 1 or 11 (Caspase 4/5 in humans), downstream of inflammasome formation. In this review, we describe the molecular events related to GSDMD-mediated pore formation. Furthermore, we summarize the so far elucidated ways of SARS-CoV-2 induced NLRP3 inflammasome formation leading to pyroptosis, which strongly contributes to COVID-19 pathology. We also explore the potential of NLRP3 and GSDMD inhibitors as therapeutics to counter excessive inflammation.

## 1. Introduction

Pyroptosis is a gasdermin-mediated, membrane pore forming, proinflammatory type of cell death. This necrotic form of cell death causes cell swelling and lysis and was initially found to be an infection triggered event occurring mostly in myeloid cells which leads to ion fluxes and release of proteins of the interleukin (IL) family. It was first described in 1992 by a group that saw a Caspase 1-dependent form of cell death in macrophages that were infected with *Shigella flexneri* [1,2] and initially thought it was a form of apoptosis because of its caspase dependency [3].

Subsequently, other groups saw similar characteristics after infection with *Salmonella typhimurium* [4]. The name pyroptosis was established in the literature in the early 2000s [5]. ‘Pyroptosis’ is derived from the Greek word ‘pyro’ owing to the fact that the proinflammatory form leads to IL-1β and IL-18 mediated inflammation such as fever; and ‘ptosis’ which means ‘falling off’, an expression commonly used for other forms of cell death. Indeed, besides uptake of cell impermeable dyes, infected cells showed typical signs of death, e.g., LDH release and phosphatidyl serine (PS) exposure to the outer leaflet. Final steps are osmotic cell swelling and rupture of the cell membrane which enables ‘alarmins’ to be set free [4,6]. The fact that lysis could be prevented by a hypertonic solution and addition of polyethylene glycols (PEG) strongly suggests that a pore-forming event leads to the observed cell death. The mechanism and molecules leading to cell permeability, however, remained unclear. It has taken over 20 years of research after the first description of pyroptosis for the effector molecules, namely, members of the gasdermin family (GSDMs) and the complex upstream events, could be identified [7,8,9,10].

Pathogen-associated molecular patterns (PAMPs) and microbe-associated molecular patterns (MAMPs) are the main upstream events of pyroptosis that lead to inflammasome formation and to Caspase 1 or Caspase 11 activation [11]. The types of inflammasomes include, e.g., the NLRP3 (NACHT, leucin-rich repeat domain (LRR), pyrin domain (PYD)), NLRC4 (NLR family CARD domain-containing 4), NLRP6 (NACHT, LRR (leucine-rich repeat), and PYD (pyrin domain) domain-containing 6), and AIM2 (absent in melanoma 2) inflammasomes. Besides PAMPs and MAMPs, intrinsic sterile events leading to danger-associated molecular pattern (DAMPs) formation can also induce inflammasome activation. In several hyperinflammatory diseases such as gout and sepsis, a dysbalance in homeostasis, e.g., by the inhibition of autophagy leading to excessive DAMP accumulation and thereby activation of inflammasomes and pyroptosis, was identified as the main cause of disease [12]. The most elucidated upstream event of pyroptosis to date is the activation of the NLRP3 inflammasome. The canonical NLRP3 inflammasome activation is composed of two activating steps: priming and trigger. During the priming step, PAMPs activate innate immune receptors that lead to NF-kB activation followed by transcription and translation of NLRP3. The freshly synthesized NLRP3 proteins accumulate in the primed cell and wait for the MAMP or DAMP-associated activation signal that leads to NLRP3 conformational change, oligomerization, ASC, and Caspase 1 recruitment. Caspase 1 then cleaves full-length Gasdermin D (GSDMD) into pore-forming mature NT-GSDM. The proforms of IL-1 and -18 are themselves cleaved by Caspase 1 into their mature forms, and released via GSDMD pores thereby leading to proinflammatory cell death. The NLRP3 inflammasome activation events are tightly regulated on post-transcriptional and post-translational levels [13]. Consequently, pyroptosis is well acknowledged as a fine-tuned process in host-cell defence. Alternatively, GSDMD and other members of the gasdermin family can also be cleaved by Caspases 3 and 8, and granzymes. There is even a bidirectional crosstalk between apoptosis and pyroptosis in monocytes and macrophages [14]. Furthermore, the role of pyroptosis and gasdermins in non-infectious diseases and cancer is emerging. These events play an important role, for example, after the application of chemotherapy when pyroptosis is mediated by the BAK/BAX-Caspase 3-GSDME pathway [15,16].

## 2. The GSDM Family

**Figure 1:**
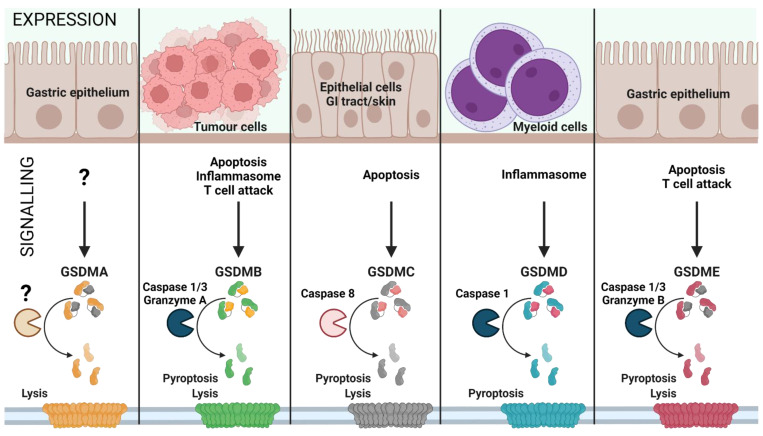
Gasdermin family: tissue-specific signalling. Gasdermin A/B/C/D/E are expressed in distinct cell types and are activated by various signals, leading to inflammatory or non-inflammatory cell lysis. The upstream events of GSDMA cleavage are not well characterized (represented as ‘?’). GSDMA downregulation in gastric epithelial cells can lead to tumour formation. Proliferating tumour cells are recognized by effector T cells that release Granzyme A, which cleaves GSDMB in cancer cells leading to pore formation and tumour cell lysis. Furthermore, GSDMB can also be activated by Caspase 1 or 3 downstream of inflammasome formation or apoptosis, leading to pyroptosis. GSDMC is activated by Caspase 8 downstream of apoptosis, linking apoptosis to pyroptosis. GSDMD is the best characterized GSDM effector molecule, cleaved downstream of inflammasome activation, leading to pyroptosis. Similar to GSDMC, GSDME links apoptosis to pyroptosis subsequent to cleavage by Caspase 3 in apoptotic cells. (Created with BioRender.com on 7 May 2022).

While GSDMD is one among five members of the gasdermin family, it is the major effector molecule of pyroptosis in myeloid cells (see Figure 1). The other members of the gasdermin family are, however, highly expressed in both benign and cancer tissues. Gasdermin A (GSDMA) is the least investigated member and is expressed in epithelial cells of the gastric tract while suppressed in gastric cancer [17,18]. Gasdermin B is highly expressed in proliferating cells and is associated with cancer development. Gasdermin C and Gasdermin E are known to switch the cell death program from apoptosis to pyroptosis; as GSDMC and GSDME are the substrates of apoptotic Caspases 8 and 3, respectively [19] (see Figure 1). In addition to GSDMA-E, Pejvakin (PJVK) has been identified as the sixth member of the gasdermin family and was characterized early as relevant in the function of auditory pathway neurons [20].

Gasdermin D is the best-studied gasdermin and we will focus this review on the events associated with GSDMD-mediated pore formation and its multiple roles during the SARS-CoV-2 (severe acute respiratory syndrome coronavirus type 2) infection and treatment.

Full-length GSDMD, the main effector of pyroptosis in macrophages, monocytes, and dendritic cells is cleaved by caspases and the effector N-terminal GSDMD domain is trafficked to the cell membrane where it oligomerizes into β-barrel transmembrane lytic pores which enable proinflammatory interleukin release and ion flux as well as trafficking of nucleotides. Cellular death follows the pyroptotic execution program. It is accepted that subsequent to the initial fluxes of ions and smaller proteins, the release of alarmins and LDH is an event after rupture since LDH is too big to escape via the pores [21,22]. Execution of pyroptotic death is tuned in a chronological manner. Pyroptotic cells show signs of intracellular death before rupturing and a study by DiPeso et al. showed that after GSDMD pore formation, ion and protein fluxes lead to the breakdown of the mitochondrial membrane potential (MMP); thus already exhibiting cell death physiology before actual lysis of the cell [23].

Pore formation, nonetheless, is not unique to pyroptosis but is a common feature of the cell death pathways—apoptosis, necroptosis, and pyroptosis—which are executed via BAX/BAK, MLKL, and GSDMs, respectively [24]. GSDMs and MLKL target the inner leaflet of the plasma membrane while BAX/BAK targets the mitochondrial outer membrane (MOM). They all have in common that the precursor forms must be (proteolytically) activated which leads to conformational changes. Necroptotic MLKL pores and BAX/BAK pores are smaller than GSDM pores. Targeting and integration into the outer membrane is executed via electrostatic interaction with the negatively charged membrane lipids as well as hydrophobic anchoring into the plasma membrane [24].

## 3. GSDMD Pore Formation

GSDMD consists of a functional 30 kDa N-terminal fragment (NT-GSDMD) which is responsible for pore formation. The auto-inhibitory C-terminal fragment is cleaved by Caspases 1, 8, and 11 (Caspases 4/5 in humans) and proteases such as neutrophil elastase or cathepsin G [25,26,27]. To localize and anchor GSDMD into the membrane, membrane-binding elements (hydrophobic anchor) and positively charged motifs bind to negatively charged membrane phospholipids [28,29]. At the membrane, NT-GSDMD oligomerization is dependent on an active Cys192 (human), Cys191 (mouse), which has to be oxidized to form a 31- to 34-fold symmetric pore, whereas GSDMA3 forms a 26- to 28-fold symmetric pore indicating size plasticity of a given gasdermin [30,31].

This oligomerization event is controlled. GSDMD oligomerization is influenced by the Ragulator-Rag-mTORC1 pathway. RagA and RagC were shown to be essential for NT-GSDMD oligomerization; where in a genome-wide CRISPR screen, RagA and RagC knock-out cells showed no pyroptosis pore formation [32,33]. It remains unclear whether RagA/RagC/GSDMD interaction takes place directly or whether intermediate partners are needed.

GSDMD has to be oxidized at several cysteine residues (Cys38, Cys56, Cys268, and Cys467) to enable pyroptosis in macrophages. A recent study suggests that this oxidation event occurs directly between ROS and these cysteines after inflammasome-induced ROS production at the mitochondria [34]. Recently, the pre-pore and pore structures were revealed with cryo-electron microscopy by the group of Hao Wu [31]. The different regulatory states before, during, and post-pore formation are now better understood on a molecular level. NT-GSDMs are β-strand pore-forming proteins characterized by high intra-strand hydrogen stability. Apart from the pore size, which is about 20–25 nm [35], the GSDMD pore acts as a negative electrostatic filter, which facilitates the release of positively charged liposome particles but hinders the release of predominantly negatively charged immature pro-IL-1 in the transmembrane passage [22]. This further explains the capability of macrophages to only release mature interleukins, even after pore formation in a so-called non-lytic state of pyroptosis [36].

Calcium^2+^ influx is described as one of the first steps after pore formation [37,38,39,40]. This ion influx leads to the activation of endosomal sorting complexes required for transport (ESCRT) of proteins I and III and calcium-dependent STING activation as well as MMP failure and loss of mitochondrial function and imbalance of the electric cell potential, which ultimately leads to pyroptotic cell death [41]. This process can be stopped in vitro by high concentration of magnesium which probably chelates nucleotide effluxes such as ATP and hinders Calcium^2+^ influx [42]. Osmotic lysis can further be prohibited by the osmoprotectant glycine [43].

Despite the fact that Caspase 1 is crucial for IL-1 release, Caspase 1 activation does not necessarily culminate in pyroptotic cell death in all cell types. Indeed, IL-1 release can be a non-pyroptotic event. Neutrophils, for example, have been observed to release IL-1β without lysis, while macrophages still undergo pyroptosis after GSDMD pore formation [44,45]. The specific regulatory events involved have not been elucidated so far. The cell adhesion protein ninjurin 1 (NINJ1) as a cell rupture mediator protein is being currently studied, as NINJ1 deficient cells do not rupture even after mitochondrial death [46].

## 4. GSDMD Post-Translational Modifications (PTM)

GSDMs oligomerization is influenced by metabolites and by post-translational modification. Although PTM prediction tools have identified many possible motifs as ubiquitination and phosphorylation sites on gasdermins, only a few modifications have been verified [47]. GSDMs are post-translationally modified mostly at cysteine residues. Investigation in the future will probably reveal more post translational modification sites, maybe targeting the so far unknown mechanisms of membrane repair after pore formation. That ESCRT-dependent membrane repair negatively regulates GSDMD has been shown by Rühl et al., but the exact mechanism of removing GSDMD pores from the membrane remains unknown [36,40]. In macrophages, GSDMD cleavage was abrogated by the binding of unsaturated dicarbonic acids at distinct cysteine residues; itaconate binding at Cys77 after prolonged LPS exposure [48] and fumarate binding at Cys191 leading to succination both result in lowered pore formation [49]. Furthermore, the succination of GSDM at Cys45 inhibits pore formation [50].

Cysteine modification can be artificially induced by drugs (e.g., necrosulfonamide, disulfiram) targeting Cys 191 and inhibiting GSDMD formation. Phosphorylation at Thr6 occurs on GSDMA and E. This modification promotes pore formation [51]. GSDME is palmitoylated through the palmitoyltransferases zDHHC2, -7, -11, and -15 at Cys407 and Cys408 [15]. This modification enables the dissociation of the NT domain from the inhibitory CT subunit after cleavage by caspases. These PTMs have been exploited in the development of therapeutics (see Section 5 below).

## 5. Pyroptosis in Non-Infectious Diseases

Pyroptosis is an important event in non-infectious diseases as well and therapeutic approaches have been developed in the hope of influencing several of these disease outcomes. The role of pyroptosis is described in sterile inflammation diseases [52], neuronal disease [53,54,55,56], cancer [57,58,59], atherosclerosis [60,61], autoimmune disease [58], acute injury [62,63], and adverse pregnancy events [64,65,66]. Even in newer roles of GSDMD, the pyroptotic effector of infectious pyroptosis has been found. GSDMD seems to be physiologically highly relevant in mucus export from goblet cells [31]. Furthermore, pyroptosis was found to be a downstream event of NETosis in neutrophils, which can be triggered by TLR or LL-37 [67,68]. Gasdermin B (GSDMB) and Gasdermin E (GSDME) are cleaved by Granzymes A and B, respectively, into their active pore-forming subunits inducing death of tumour cells by pyroptosis and resulting in local inflammation [57,69]. Granzyme A from cytotoxic T-cells cleaves GSDMB in the tumour cells at lysine 229 and 244 leading to pore formation in target cells [69]. Liu et al. showed that the cytokine release syndrome is possibly caused due to granzyme B activation by Caspase 3 which causes GSDME pyroptosis in target cells. The pyroptosis of the target cells leads to Caspase 1 triggered macrophage pyroptosis which, in turn, leads to cytokine release from myeloid cells and to cytokine release syndrome [59].

## 6. GSDMs as Therapeutic Targets

With the elucidation of the effectors of the pyroptosis pathway and their role in disease, the search for inhibitors has become a highly investigated field. The majority of promising drugs such as disulfiram and necrosulfonamide (NSA) target reactive cysteine residues because of their critical role in cytosol gasdermin recruitment and pore formation [70] (see Table 1). Hu et al. characterized disulfiram, an FDA-approved drug for treating alcohol addiction as a potent LPS-induced sepsis inhibitor in mice. At nanomolar concentrations, disulfiram covalently modifies human Cys191 in GSDMD to block pore formation while allowing IL-1β and GSDMD processing [71]. Punicalagin, a polyphenol, probably prevents NT-GSDMD cytoplasmic membrane insertion through its antioxidative effect on reactive thiols [72] (see Table 1). Upstream inhibition of GSDMD pore formation takes place through inhibitors such as Bay 11–7082, an identified inhibitor of NF-κB, or z-VAD-fmk, a Caspase 1 specific inhibitor [73,74] (see Table 1). Natural modified metabolites such as unsaturated dicarbonic acids dimethlylfumarate (DMF) inactivate GSDMD and GSDME by succination at Cys101 and Cys45. DMF use in the treatment of neurological disorders such as multiple sclerosis (MS) is currently being evaluated [50,75] (see Table 1). 2-Bromopalmitate was found to inhibit the CT-GSDME palmitoylation and prevent chemotherapy-induced GSDME-mediated pyroptosis [15].

Due to the existence of many reactive thiols in the inflammatory pathways, the unspecific nature of all these drugs could likely be an issue with regard to off-target effects. More studies have to be conducted to characterize the mode of action of these compounds in vivo.

It has been found that treatment failure of aggressive HER2 positive breast cancers is associated with the coamplification and coexpression of an *Erb2* neighbour gene, namely, the *GSDMB* gene [79] (see Table 1). An approach independent of small molecules to prevent pyroptosis was published by Molina-Crespo et al. where an anti-GSDMB antibody was intracellularly delivered into HER2 positive breast cancer cells, proving that protumour GSDMB functions such as migration, metastasis, and therapy resistance could be reduced [76]. This promising approach should be further evaluated for translational potential. Although many attempts have been made to inhibit pyroptosis in order to treat inflammation, most approaches have not reached drug approval and the definition and inhibition of targets during pyroptosis needs further elucidation.

## 7. SARS-CoV-2 Triggered NLRP3-Mediated Pyroptosis

With the rapid worldwide spread of the novel coronavirus (SARS-CoV-2) in late 2019; the World Health Organization (WHO) declared a global emergency. Since then, the pandemic has had a significant toll on human health and the world economy. Globally, to date there have been 497,057,239 confirmed cases of COVID-19, including 6,179,104 deaths, reported to WHO [https://covid19.who.int/ (accessed on 12 April 2022)].

SARS-CoV-2 is an enveloped RNA virus that is composed of several proteins: the nucleocapsid, the matrix, the envelope, and the spike [80] . It is transmitted from person to person primarily through droplet and aerosol routes. COVID-19 manifests most commonly as a respiratory illness with hyperinflammation of the lung in patients with severe disease [81] . Consequently, clinicians have started to investigate whether and how inflammasome activation and pyroptosis are linked to COVID-19 symptoms. Understanding the connection between SARS-CoV-2 infection and pyroptosis-mediated inflammation has been critical since the emergence of the pandemic, and even more so because an inflammasome and pyroptosis-mediated inflammatory signature could present an opportunity for therapeutic intervention in which pyroptosis-associated events are targeted. In fact, it has been shown that SARS-CoV-2 infection leads to NLRP3 inflammasome activation in vitro and in vivo [82,83]. Inflammasome activation was also associated with COVID-19 severity [82,83] and NLRP3 activation might even be a suitable predictor of COVID-19 disease severity and a potential therapeutic target. Indeed, inhibition of the NLRP3 inflammasome with the well-characterized NLRP3 inhibitory compound MCC950 reduced COVID-19 pathology in mice [84,85,86].

**Figure 2:**
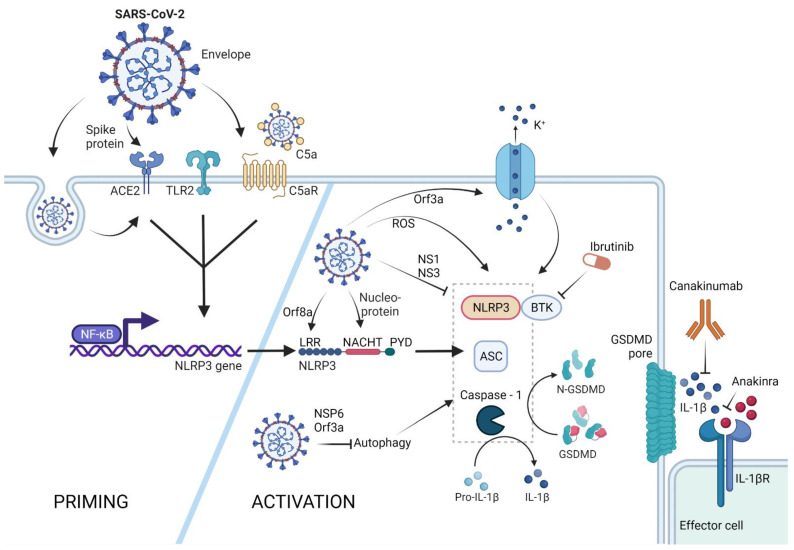
NLRP3 inflammasome activation by SARS-CoV-2. The NLRP3 inflammasome can be primed by SARS-CoV-2 proteins spike and envelope, and in its opsonized form by activating the ACE-2, TLR-2, and complement receptors, respectively. SARS-CoV-2-infected cells upregulate ACE-2 receptor expression, rendering these cells more sensitive to SARS-CoV-2 response. Activation of these receptors leads to NFκB-mediated *Nlrp3* gene transcription and translation. The NLRP3 inflammasome can form in response to ROS generation in SARS-CoV-2-infected cells, via potassium efflux through SARS-CoV-2 Orf3a-generated pores, or via direct interaction with the SARS-CoV-2 proteins orf8a and nucleoprotein. SARS-CoV-2 is capable of NLRP3 inflammasome inhibition via NS1 and NS13 proteins, as well as through inhibition of autophagy, a general NLRP3 trigger. Therapeutic potential lies in the inhibition of the effector molecule IL-1β via canakinumab or anakinra, as well as inhibition of the NLRP3 accessory protein BTK by ibrutinib. (Created with BioRender.com on 13 April 2022).

Furthermore, experimental treatment of severe COVID-19 with canakinumab, an anti-IL-1β antibody, showed some beneficial effects early into the pandemic; similar to that of compassionate use of remdesivir, a viral polymerase inhibitor [77,87] (see Figure 2).

Similar results were also observed after anakinra treatment, which blocks the IL-1 receptor thus inhibiting downstream signalling [78,88,89] (see Figure 2).

The promising effect of anakinra treatment was also confirmed in a phase III clinical trial which showed significant reduction of COVID-19-related mortality when severe patients were treated with anakinra at early stages of the disease [90].

The observation of NLRP3 inflammasome activation in vivo in COVID-19 patients and subsequent studies treating severe patients with experimental anti-pro-inflammatory cytokines allowed for the underlying molecular mechanisms to also be studied.

First of all, a landmark study conducted RNAseq on lung tissues from COVID-19-affected humans and compared the transcriptome with that of healthy lung donors. It was shown that NLRP3 signalling is upregulated in COVID-19 lungs, together with many ‘DAMPs’ such as metabolic dysregulation and ROS that are triggered by SARS-CoV-2 and can lead to NLRP3-inflammasome-mediated pyroptosis leading to a cytokine storm[91] . After this significant indication that SARS-CoV-2 promoted NLRP3 activation [91], besides the generic infection-associated signals, specific SARS-CoV-2 viral particle-mediated NLRP3 inflammasome activation has also been described on multiple levels.

Some knowledge derived from studies with SARS-CoV could be taken as indicative for the connection between SARS-CoV-2 and NLRP3. For example, it had been shown that SARS-CoV Orf3a could directly bind TRAF3 as well as ASC and activate the NLRP3 inflammasome [92] (see Figure 2).

Orf3a was able to induce both priming and activation signals of the NLRP3 inflammasome and lead to pyroptosis and mature IL-1β release. Although Orf3a belongs to the Viriporin pore-forming SARS-CoV protein family, SARS-CoV Orf3a-triggered NLRP3-mediated pyroptosis seemed to be independent of its pore-forming activity [93]. The role of Orf3a in NLRP3 inflammasome activation was also investigated in the case of SARS-CoV-2. Interestingly, SARS-CoV-2 Orf3a was also found to induce NLRP3 inflammasome activation. However, this was based on the pore-forming activity of Orf3a [94]. The discrepancies between the two mechanisms need to be further explored (see Figure 2).

The Orf8b protein of SARS-CoV-2 was shown to be deadly on multiple levels. In non-myeloid cells that lack the NLRP3 inflammasome machinery, Orf8b accumulates and leads to ER stress, lysosomal damage, and caspase-independent cell death. However, in myeloid cells carrying NLRP3, it directly interacts with the NLRP3 LRR domain leading to ASC-speck formation and pyroptosis [95]. Furthermore, it was found that the nucleocapsid of SARS-CoV-2 also binds directly to NLRP3 and leads to inflammasome activation and pyroptosis [96] (see Figure 2).

The non-structural protein NSP6 was also found to be a strong NLRP3 and pyroptosis initiator. NSP6 was shown to inhibit the lysosome–autophagosome system of the cell, which is known to trigger NLRP3 inflammasome formation [97] (See Figure 1).

Orf3a was also associated with inhibition of autophagy by VPS39 interaction, highlighting another pathway of NLRP3 activation by Orf3a, in addition to pore formation and potassium efflux (see Figure 2).

However, the NLRP3 assembly trigger signal is not only associated with SARS-CoV-2. It was recently shown that the envelope protein of SARS-CoV-2 is a TLR2 ligand, and TLR2-SARS-CoV-2 envelope protein engagement leads to effective NLRP3 inflammasome priming. Blocking TLR2 during coronavirus infection thus leads to reduced inflammation [98] (see Figure 2).

SARS-CoV-2 infection manifests in neurological symptoms as well, which also seems to precipitate Parkinson´s disease [99] according to a preprint publication that suggests that the SARS-CoV-2 spike protein activates the NLRP3 inflammasome ex vivo via ACE-2 interaction in human microglia. Additionally, a-synuclein, which is the underlying aggregate causing Parkinson´s disease, significantly enhanced inflammasome activation, which was completely dependent on NLRP3. The NLRP3-mediated pyroptosis in microglia might explain COVID-19-driven neuroinflammation [100]. This study, however, was not the first one to connect NLRP3 inflammasome priming and the SARS-CoV-2 spike protein. It was shown that the spike protein primes the NLRP3 inflammasome and leads to pyroptosis and IL-1β secretion ex vivo in cells derived from SARS-CoV-2-experienced individuals although not from healthy donors [101]. Accordingly, SARS-CoV-2 infection renders macrophages in a distinct proinflammatory state that makes the spike protein even more immunogenic towards these immune cells. Interestingly, a different study showed that healthy monocytes do not express the ACE-2 receptor, while SARS-CoV-2-experienced monocytes express it in low levels, highlighting tissue-specific differences and SARS-CoV-2-mediated NLRP3 inflammasome activating pathways. It might very well be possible that the ACE-2 expression of SARS-CoV-2-experienced monocytes enables spike-specific NLRP3 inflammasome priming that was observed in the above study [102]. SARS-CoV-2 infection of monocytes and macrophages is not necessarily dependent on spike-ACE-2 interaction. It can be Fc-receptor or complement-receptor mediated, or via antibody and complement opsonized virus [103,104] (see Figure 2).

Studying the SARS-CoV-2 genome itself provided further evidence that SARS-CoV-2 infection indeed leads to NLRP3 inflammasome activation: a cDNA screen of 28 SARS-CoV-2 ORFs revealed that the proteins NS1 and NS13 inhibit NLRP3 inflammasome activation and pyroptosis-mediated IL-1β release—a function which the virus would not invest in unless it posed a threat to its infectivity [105].

As accumulating evidence supports the role of NLRP3 in the development of SARS-CoV-2-mediated inflammatory condition, targeting NLRP3 directly instead of the pyroptosis-related proinflammatory cytokines is emerging as a therapeutic strategy. Colchicine is an NLRP3 inhibiting molecule, although the exact underlying mechanism is not fully understood [106]. Regardless, several clinical trials have been conducted to evaluate the beneficial effect of colchicine treatment in early SARS-CoV-2 infection [107].

As the condition “long COVID-19” is also associated with a prolonged inflammatory state, an extended NLRP3 treatment period should also be considered.

Furthermore, after the identification of an FDA-approved inhibitor of the NLRP3-specific regulatory protein Bruton´s tyrosine kinase (BTK), the application of the commercially available BTK inhibitor ibrutinib in severe COVID-19 cases was investigated. First of all, a study showed that Waldenström Macroglobulinemia patients (a form of BTK-mediated B cell malignancy) that received ibrutinib cancer treatment had beneficial outcomes when contracting COVID-19, especially patients on high-dose ibrutinib treatment [108]. A second study used off-label acalabrutinib (an ibrutinib-like BTK inhibitor) in severe COVID-19 patients which significantly increased the lung function of the patients [109]. Although a direct link between NLRP3-mediated pyroptosis and ibrutinib treatment has not been established yet, the promising results call for further investigation of a broader ibrutinib treatment of COVID-19 patients (see Figure 2).

Although increasing evidence supports the role of SARS-CoV-2-induced NLRP3 activation and pyroptosis in COVID-19 disease manifestation, one study showed that NLRP3 depletion or impaired pyroptosis had a negative effect on murine coronavirus infection outcomes in mice [110].

However, as this study was conducted with a murine coronavirus MHV (mouse hepatitis virus), a direct comparison to human SARS-CoV-2 may be difficult.

## 8. Summary and Perspective

Pyroptosis is a key cell response to pathogen invasion, as well as to sterile danger. Many events on a cellular and molecular level upstream and downstream of the activation of the key pyroptosis effector molecule, GSDMD, have been characterized. Researchers are eager to understand the exact mechanism of this inflammatory cell death, as many inflammatory conditions could benefit from pyroptosis inhibition. One of these, and the most current threat, is COVID-19 disease caused by the β-coronavirus SARS-CoV-2 that emerged in 2019 and caused a devastating pandemic. Several attempts have been made to dampen COVID-19-associated lung pathology by administering NLRP3 (through the BTK inhibitor ibrutinib) or IL-1β inhibitory molecules (anakinra or canakinumab). These showed promising effects, however, further investigation and analysis of these treatment options is necessary.

## Figures and Tables

**Table 1:** Cell lysis and pyroptotic pathway inhibitors.

Target	Agent	Mode of Action	Citation
NF-κB	Bay 11–7082	Upstream inhibition of NLRP3 priming	[73]
Caspase 1	z-VAD-fmk	Inhibition of GSDMs and pro-IL-1β cleavage	[74]
GSDMB	Anti-GSDMB antibody	Direct binding of GSDMB, thereby reducing tumour growth and distant metastasis in HER2 positive cancer	[76]
GSDMD	Disulfiram	Inhibits assembly of NT-GSDMD by binding at Cys191	[71]
	Dimethylfumarate (DMF)	DMF inhibits GSDMD and GSDME at Cys45 and Cys191 and reduces demyelination in MS	[75]
	Necrosulfonamide (NSA)	Inhibits formation of NT-GSDMD by binding at Cys191	[70]
	Punicalagin	Probably inhibits GSDMD insertion into cell membrane	[72]
GSDME	2-Bromopalmitate (2-BP)	Counteracts the CT-GSDME palmitoylation; prevents chemotherapy-induced GSDME-mediated pyroptosis	[15]
IL-1β	Canakinumab	Binds and neutralizes IL-1β	[77]
IL-1βR	Anakinra	IL-1βR antagonist	[78]

## Data Availability

Not applicable.
